# Student Pharmacists’ Perspectives of In-Person versus Virtual Research Poster Presentations

**DOI:** 10.3390/pharmacy10050104

**Published:** 2022-08-26

**Authors:** David R. Axon, Megan Whaley

**Affiliations:** Department of Pharmacy Practice and Science, University of Arizona College of Pharmacy, Tucson, AZ 85721, USA

**Keywords:** posters, student pharmacists, research course, presentations, interviews

## Abstract

This study assessed the preferences of fourth-year student pharmacists for an in-person versus virtual pharmacy research poster session. An electronic survey was administered to all fourth-year student pharmacists enrolled in a research project course in Fall 2021 (*n* = 132). Six items explored students’ opinions towards research posters using a five-point agreement scale. Twelve items explored students’ preferences for either research poster format. Students also indicated their overall preference for an in-person or virtual research poster session. Data were analyzed descriptively. A total of 63 fourth-year student pharmacists completed the questionnaire. The median agreement score was four out of five, indicating favorable attitudes towards the importance of research posters in pharmacy curriculum. Most students said they would enjoy research posters more, and would be more able to present at, participate in and ensure that all can participate in poster sessions if the poster sessions were virtual as opposed to in-person. Most (76.2%) students indicated a preference for virtual rather than in-person research poster sessions. In conclusion, the study results suggest that student pharmacists prefer virtual rather than in-person poster sessions. Further research is recommended to explore the comparative effectiveness of these poster formats to achieve learning outcomes in varying university pharmacy programs.

## 1. Introduction

The research poster project is considered an essential component of many project and team-based healthcare curriculums [1,2,3,4]. The American College of Clinical Pharmacy (ACCP) recommends that all professional pharmacy curriculums should train student pharmacists to effectively communicate clinical research to other healthcare professionals and patients [5]. Outcomes associated with research poster projects include improvements in the following competencies: public speaking [1,4], interprofessional and peer collaboration [1,6], community engagement [1], accumulation of evidence [1,4,6], confidence [4,6], organization of data into visual displays [1,4], professional writing [1,6], and composition of abstracts [4,6]. In addition, Morbitzer et al. recently reported that applicants who had presented a poster at a national meeting during their pharmacy program were more likely than those who had not to be offered an interview for a health-system pharmacy administration residency program (80% versus 60%, *p* = 0.02) [7].

Every year, conferences all over the world request student research abstracts, offering students the chance to gain those transferable skills [8,9,10]. Yet, among research programs which offer training in research poster presentation, some report that costs associated with in-person poster sessions and conferences are prohibitive and inflexible [11,12,13,14]. Many have called for the consideration of virtual posters for their convenience [15], creative flexibility [15,16,17,18], multimedia capacity [15,17,18], and inclusivity [15,19].

In 2020 and 2021, many major pharmacy conferences were held virtually with no in-person components due to the coronavirus disease 2019 (COVID-19) pandemic [14,20,21]. In addition, research suggests that blended learning in some pharmacy coursework is more effective at achieving learning outcomes than traditional in-person formats [22]. However, recent research also suggests that some university post-COVID shifts to blended learning have had inequitable and unacceptable impacts on university students’ wellbeing [23]. Given that the virtual format for conferences and research poster sessions may continue in future, there is a need for students to develop their virtual presentation skills so that they are appropriately prepared to participate in future virtual research poster sessions. Students may have little or no experience presenting a research poster, yet colleges of pharmacy that offer research projects within the curriculum may have to choose whether to host an in-person or virtual research poster session for their students. However, limited research has been done to explore student pharmacists’ preferences for an in-person versus virtual research poster session. Therefore, the objective of the present study was to assess student pharmacists’ preferences for an in-person versus virtual research poster session based on their perceptions of how effective, enjoyable, and straightforward they think these formats would be. A secondary objective was to assess student pharmacists’ perceptions of their confidence with research posters and the value of the research poster in their pharmacy education.

## 2. Methods

### 2.1. Doctor of Pharmacy Research Curriculum Overview

All student pharmacists at the University of Arizona R. Ken Coit College of Pharmacy are required to complete a capstone research project as part of their curriculum. Initially, students complete six credits of drug literature evaluation coursework, including statistical methodology and study design, in their first and second year. Additionally, students complete three credits of quality improvement (QI) coursework that includes a year-long, team-based QI project and presentation of their findings at a poster session at the end of the second year. In the third and fourth year, the capstone research project is facilitated by a series of three two-credit courses. In the spring semester of the third year, students participate in a proposal writing course where they design their study and obtain institutional review board (IRB) approval (if necessary). During the fourth year, students receive credit for collecting and analyzing data in the fall and writing a research report and presenting a research poster in the spring. Historically, the poster session was held in-person, with students attending an all-day poster session to present their work to peers and faculty advisors. Students received participation points for visiting their peers’ posters and discussing their findings. For the graduating classes of 2020 and 2021, the research poster session was held virtually due to the COVID-19 pandemic. The virtual format required students to record a presentation of their poster and provide a link for their peers to review and comment on during the research poster session. Students received participation points for making at least 25 comments on their peers’ posters. All three courses are coordinated and taught by an academic pharmacist faculty member with a PhD in pharmacy administration. Student pharmacists work independently or in teams under the supervision of their advisor(s) to design and complete their research project.

### 2.2. Study Eligibility and Design

Fourth-year student pharmacists enrolled in the University of Arizona R. Ken Coit College of Pharmacy research course in Fall 2021 (*n*= 132) were eligible to participate in this survey study.

### 2.3. Instrument Development

An initial draft of the questionnaire was developed with several rounds of revisions until the instrument was deemed to have appropriate face validity by the research team. The final instrument was hosted in Research Electronic Database Capture (REDCap) (Version 12.0.0, Nashville, TN, USA) and contained 25 items (Appendix A).

The first section contained six items that explored student pharmacists’ opinions and attitudes towards pharmacy research posters using a five-point Likert scale (strongly disagree, disagree, neutral, agree, strongly agree). The first three items explored whether respondents thought developing a research poster, presenting a research poster, and participating in a research poster session were valuable elements of their pharmacy curriculum. The second three items asked about how confident they were in their ability to develop a poster, present a poster, and participate in a research poster session.

The second section included 12 items exploring student pharmacists’ preferences towards in-person versus virtual research posters. Respondents were asked to select one of three preferences for each item: in-person, virtual, or no difference. The first set of three items asked which format students thought would facilitate their acquisition of more research poster development skills, poster presentation skills, and poster session participation skills. The second set of three items asked which format student pharmacists thought they would enjoy the most if they were developing a research poster, presenting a research poster, and participating in a research poster session. The third set of three items asked which format students thought was most feasible for them if they were developing a research poster, presenting a research poster, and participating in a research poster session. The fourth set of three items asked which format students thought would be the most effective way to communicate findings from a research project, to critically evaluate findings from a research project, and to help ensure everyone can participate in the research project poster session. Lastly, one item asked students to indicate their overall preference for in-person or virtual research poster session (there was no “no difference” option), and one open response item for any comments about research poster preferences.

The instrument concluded with five demographic/descriptive items: sex (male, female), campus (Tucson, Phoenix), previously worked on a research project (not including QI project; yes, no), worked independently or as part of a group on the research project (independent, group), and whether they anticipate holding a position that will involve conducting research following graduation from the pharmacy program (yes, no).

### 2.4. Data Collection

Data were collected over a three-week period in November 2021. This time was chosen because students were midway through their capstone research projects, and thus appropriately familiar with the work involved. All eligible participants were sent an email that included background information about the study and a link to participate in the online questionnaire. Reminder emails were sent after one and two weeks. Students had been informed of the two different approaches (i.e., in-person versus virtual) during their third-year proposal writing course and advised that a decision on the modality for their poster session would be determined during their fourth year. Students were not offered any incentive to participate in this study.

### 2.5. Data Analysis

Data were analyzed descriptively using Microsoft Excel (Version 16.57, Redmond, WA, USA). Descriptive statistics including frequencies (with percentages) and medians (with interquartile ranges) were calculated as appropriate.

## 3. Results

A total of 63 fourth-year student pharmacists completed the questionnaire (47.7% response rate). A majority were female (66.7%), attended the Tucson campus (77.8%), had not worked on a research project before beyond their second-year quality improvement project (60.3%), were working as part of a group (95.2%), and did not anticipate holding a position that would involve conducting research following graduation from the pharmacy program (69.8%) (Table 1).

**Figure 1:**
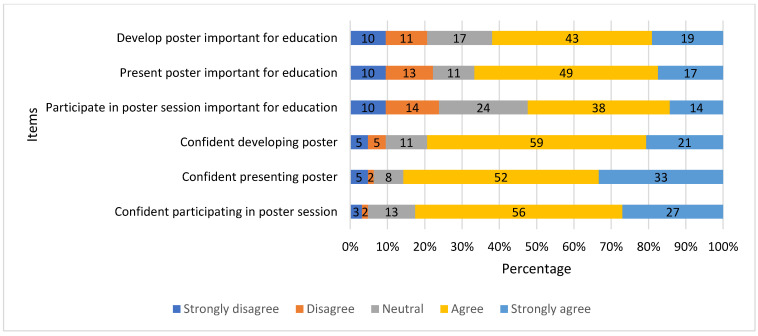
Level of agreement among fourth-year student pharmacists who completed the survey about pharmacy research posters.

The median agreement score was four out of five for items addressing students’ perceived importance of (1) developing, (2) presenting, and (3) participating in a research poster session. Additionally, the medium agreement score was four out of five for items addressing student pharmacists’ confidence in their ability to (4) develop, (5) present, and (6) participate in a research poster session. The interquartile range was 1 for all items except confidence in ability to develop a research poster, which was 0. The percentage of students who responded to each level of agreement for each item are shown in Figure 1.

**Figure 2:**
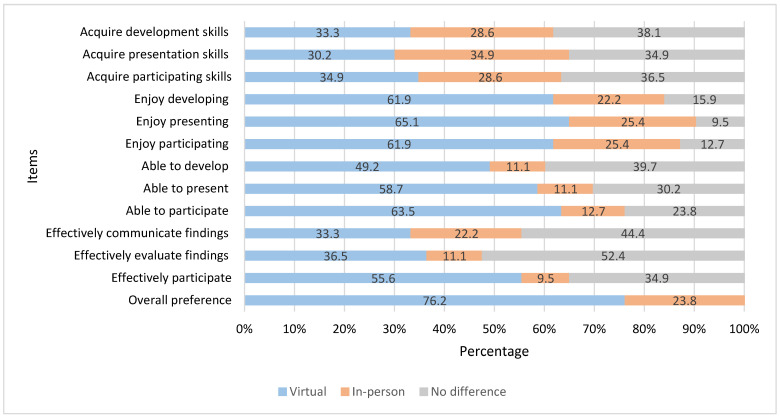
Student pharmacists’ preferences for a virtual versus in-person research poster session. Figure legend: **Acquire development skills**: I would acquire more poster development skills if the research poster session is. **Acquire presentation skills**: I would acquire more poster presentation skills if the research poster session is. **Acquire participating skills**: I would acquire more skills participating in the poster session if the research poster session is. **Enjoy developing**: I would most enjoy developing a research poster if the research poster session is. **Enjoy presenting**: I would most enjoy presenting a research poster if the research poster session is. **Enjoy participating**: I would most enjoy participating in a research poster session if the research poster session is. **Able to develop**: I would be most able to develop a research poster if the research poster session is. **Able to present**: I would be most able present a research poster if the research poster session is. **Able to participate**: I would be most able to participate in a research poster session if the research poster session is. **Effectively communicate findings**: The most effective way to communicate findings from a research project is. **Effectively evaluate findings**: The most effective way to critically evaluate findings from a research project is. **Effectively participate**: The most effective way to help ensure everyone has the opportunity to participate in the research project poster session is. **Overall preference**: Overall, if I had to choose one approach for developing a research poster, presenting a research poster, and participating in a research poster session, I would choose.

Student pharmacists most commonly endorsed “no difference” in their perceived ability to acquire research poster skills using a specific format, with 38.1% indicating no difference in their ability to acquire developmental skills, 34.9% indicating no difference in their ability to acquire presentation skills, and 36.5% indicating no difference in their ability to acquire participating skills. Most student pharmacists reported that they would enjoy developing (61.9%), presenting (65.1%) and participating (61.9%) in a poster session more if it was virtual rather than if it were in-person. Most agreed that if the posters were virtual, they would be most able to present (58.7%) and participate (63.4%) in a poster session. A plurality agreed that they would be most able to develop a poster if it were virtual (49.1%). Most agreed that virtual posters would be a more effective format for ensuring all can participate in poster sessions (55.6%), while if they had to choose a format, more than three quarters (76.2%) endorsed a preference for the virtual format over an in-person format. There were no items on which most respondents endorsed in-person research posters as their preferred format (Figure 2).

## 4. Discussion

This paper explored the preferences of fourth-year student pharmacists completing a research project at The University of Arizona R. Ken Coit College of Pharmacy for an in-person versus virtual research poster session to be held at the end of their pharmacy curriculum. The first key finding from this study was that student pharmacists preferred a virtual research poster session rather than an in-person research poster session. The second key finding was that student pharmacists agreed that a research poster session is an important part of their pharmacy curriculum, and perceived they were confident in their ability to develop, present and participate in a research poster session. Both key findings are discussed further below.

Interestingly, in addition to more than three quarters of students endorsing a preference for virtual poster presentations over in-person presentations, there was no instance where a majority preferred the in-person format. The preference for virtual formats was most pronounced when asked which format they would enjoy most, be able to present in, be able to participate in poster sessions and effectively ensure all students can participate in the poster sessions. One recent study compared digital versus printed posters for a pharmacy course and found that students significantly preferred digital posters compared to printed posters (68.5% vs. 31.5%, *p* < 0.05) [14]. Our finding corroborates this existing knowledge, although additional studies at different schools of pharmacy are needed before any more general conclusions can be drawn. Our findings are interesting given that the majority of students in this class were based at the main (Tucson) campus where the in-person poster session would have been held, although some students are based at the Phoenix campus and would have to have travelled to the Tucson campus for the in-person session. Additionally, most of their coursework since the start of the COVID-19 pandemic in March 2020 has been virtually delivered, with limited in-person learning opportunities. Therefore, these student pharmacists’ exposure to virtual presentations may have engendered confidence in their skillful use of them. Bashir et al. recently explored the shifts in preferences around virtual learning among students experiencing university transitions to online learning platforms during the COVID-19 pandemic [23]. The results of their survey suggest that although most students were successful in making the transition to online learning, they also experienced various detriments to their sleep, concentration, mental health and access to adequate home and workspaces. Therefore, students may be experiencing a range of uncaptured effects from the transition to online learning that were not captured in our survey yet may explain preferences for virtual poster formats.

A plurality of respondents agreed that they believed there was no difference between which format best ensured their acquisition of research poster skills. A plurality also agreed that they perceived there was no difference between formats in how effectively they could communicate or evaluate the research. Student indifference towards a preferred format for skill acquisition may be explained by the fact that these students experienced their year two quality improvement research poster presentation in the virtual format amidst COVID-19 pandemic related, university-wide remote instruction, and a majority (60.3%) had not experienced a research project beyond their virtual quality improvement project. Recently it has been suggested that in-person (printed) poster sessions lack flexibility and that alternative methods to improve the availability of poster sessions should be considered [11]. A virtual poster presentation, as favored by student pharmacists in this study, may be one such alternative.

The second key finding was that student pharmacists typically agreed (as indicated by a median score of four out of five on an agreement scale) that developing a research poster, presenting a research poster, and participating in a research poster session is an important part of their pharmacy curriculum. Likewise, student pharmacists typically agreed (also indicated by a median score of four out of five on an agreement scale) that they were confident in their ability to develop a research poster, present a research poster, and participate in a research poster session. One explanation for this result is that these student pharmacists have already received at least two years of research training in their pharmacy curriculum prior to responding to this survey. Henchey et al. recently found that student pharmacists experienced statistically significant increases in confidence in their ability to conduct a wide range of research activities following a Doctor of Pharmacy capstone research project [6]. Additionally, previous research, focused on teamwork, found that students at this institution endorsed positive attitudes towards teamwork during their second-year quality improvement projects [24]. Positive prior experiences with research projects may inform perceptions of their capstone research projects [24].

Limitations of this study include difficulty validating the accuracy of responses, small sample size, 48% response rate, and lack of a control group. A response bias may be present where the agreement that research projects are important and confidence in their ability to conduct research is enhanced among those who are interested in participating in a survey on research projects. Prior training in research may have influenced respondents’ positive attitudes towards research. The survey did not specifically assess if students had participated in a prior research poster, instead asking if they had prior research experience. In addition, these students have received their instruction primarily in the virtual format since March 2020, the start of the COVID-19 pandemic. Therefore, preferences for virtual posters may reflect a shift in competencies students have gained for virtual formats. The findings, therefore, may not be generalizable to other populations of student pharmacists. Future studies using a larger sample of student pharmacists from other colleges of pharmacy would help improve the external validity of the findings and may offer opportunities for additional analyses. In addition, future research is needed to compare students’ preferences for in-person versus virtual research poster sessions after they have had the opportunity to experience both formats, so that students were more experienced when stating their preferences. There are limited published data about attendance and engagement in virtual research poster sessions as compared to in-person research poster sessions, although anecdotal evidence suggests fewer people visit virtual posters than in-person posters. In these courses, participation in the poster session was encouraged by awarding points to students for visiting and commenting on their peers’ posters. However, further research may be warranted to investigate if and why there are differences in the number of viewers between virtual and in-person research poster sessions. Finally, further research could be conducted to explore the reasons why students indicated a preference for one modality over another, perhaps using qualitative techniques (e.g., interviews or focus groups).

## 5. Conclusions

The first key finding from this study of student pharmacists (who experienced a predominantly virtual curriculum due to COVID-19) at one college of pharmacy was that students preferred a virtual research poster format rather than an in-person research poster format. The second key finding was that student pharmacists agreed that a research poster project is an important part of their pharmacy curriculum and are confident in their ability to develop posters, present posters and participate in a research poster session. The findings from this study suggest that student pharmacists prefer virtual to in-person poster formats. Future research is recommended to explore the comparative effectiveness of these poster formats to achieve learning outcomes. Further research is recommended at additional universities to explain student pharmacists’ preferences for poster formats in the changing learning environments created by the COVID-19 pandemic.

## Figures and Tables

**Table 1:** Demographic and descriptive characteristics of fourth-year student pharmacists who completed the survey about pharmacy research posters.

Descriptive Items	N (%)
Sex	
Female	42 (66.7)
Male	21 (33.3)
Which University of Arizona campus do you attend?	
Tucson	49 (77.8)
Phoenix	14 (22.2)
Have you worked on any other research project (other than the Quality Improvement project) before your Doctor of Pharmacy research project?	
Yes	25 (39.7)
No	38 (60.3)
Are you working independently or as part of a group for your Doctor of Pharmacy research project?	
Independent	3 (4.8)
Group	60 (95.2)
Following graduation from the Doctor of Pharmacy program, do you anticipate holding a position that will involve conducting research?	
Yes	19 (30.2)
No	44 (69.8)

## Data Availability

The data presented in this study are available on request from the corresponding author.

## Appendix A

**Table A1:** Research poster questionnaire administered to fourth year student pharmacists enrolled in a capstone research project course.

Please Rate Your Level of Agreement with the Following Statements about Research Posters:	Strongly Disagree	Disagree	Neutral	Agree	Strongly Agree
**Developing** a research poster is an important part of my Doctor of Pharmacy education	◯	◯	◯	◯	◯
**Presenting** a research poster is an important part of my Doctor of Pharmacy education	◯	◯	◯	◯	◯
**Participating** in a research poster session is an important part of my Doctor of Pharmacy education	◯	◯	◯	◯	◯
I am confident in my ability to **develop** a research poster	◯	◯	◯	◯	◯
I am confident in my ability to **present** a research poster	◯	◯	◯	◯	◯
I am confident in my ability to **participate** in a research poster session	◯	◯	◯	◯	◯

Please indicate your preference for the following statements about research posters:			Virtual	In Person	No Difference
I would acquire more poster **development** skills if the research poster session is:			◯	◯	◯
I would acquire more poster **presentation** skills if the research poster session is:			◯	◯	◯
I would acquire more skills **participating** in the poster session if the research poster session is:			◯	◯	◯
I would most enjoy **developing** a research poster if the research poster session is:			◯	◯	◯
I would most enjoy **presenting** a research poster if the research poster session is:			◯	◯	◯
I would most enjoy **participating** in a research poster session if the research poster session is:			◯	◯	◯
I would be most able to **develop** a research poster if the research poster session is:			◯	◯	◯
I would be most able **present** a research poster if the research poster session is:			◯	◯	◯
I would be most able to **participate** in a research poster session if the research poster session is:			◯	◯	◯
The most effective way to communicate findings from a research project is:			◯	◯	◯
The most effective way to critically evaluate findings from a research project is:			◯	◯	◯
The most effective way to help ensure everyone has the opportunity to participate in the research project poster session is:			◯	◯	◯
Overall, if I had to choose one approach for developing a research poster, presenting a research poster, and participating in a research poster session, I would choose:			◯	◯	
Please provide any comments you have about virtual or in-person posters:					

Please enter the following demographic and background information:					
Sex	Male		◯	Female	◯
Which University of Arizona campus do you attend?	Tucson		◯	Phoenix	◯
Have you worked on any other research project (other than the Quality Improvement project) before your Doctor of Pharmacy research project?	Yes		◯	No	◯
Are you working independently or as part of a group for your Doctor of Pharmacy research project?	Independent		◯	Group	◯
Following graduation from the Doctor of Pharmacy program, do you anticipate holding a position that will involve conducting research?	Yes		◯	No	◯

## References

[1] Kelsch M.P., Werremeyer A.B. (2011). Poster project to emphasize public health in the pharmacy curriculum. Am. J. Pharm. Educ..

[2] Karimi R., Meyer D., Fujisaki B., Stein S. (2014). Implementation of an integrated longitudinal curricular activity for graduating pharmacy students. Am. J. Pharm. Educ..

[3] Accreditation Council for Pharmacy Education. https://www.acpe-accredit.org/pdf/Standards2016FINAL.pdf.

[4] Kelly M.M., Blunt E., Nestor K., Mondillo J. (2020). Professional conference poster presentation: Innovative professional development assignment in nurse practitioner education. J. Nurs. Educ..

[5] Lee M.W., Clay P.G., Kennedy W.K., Kennedy M.J., Sifontis N.M., Simonson D., Sowinski K.M., Taylor W.J., Teply R.M., Vardeny O. (2010). The essential research curriculum for Doctor of Pharmacy degree programs. Pharmacotherapy.

[6] Henchey C., Keefe K., Munger M.A., Witt D.M. (2020). Fostering PharmD skills related to research and quality improvement through mentored projects. Am. J. Pharm. Educ..

[7] Morbitzer K.A., Eckel S.F. (2019). Association between student characteristics and rankings when applying for a residency in health-system pharmacy administration. Am. J. Health Syst. Pharm..

[8] The Pediatric Pharmacy Association. https://www.ppag.org/index.cfm?pg=CallforAbstracts.

[9] International Society for Pharmacoeconomics and Outcomes Research. https://www.ispor.org/conferences-education/conferences/submit-abstract/abstract-submission-instructions.

[10] American Society of Health-System Pharmacists. https://midyear.ashp.org/posters/students?loginreturnUrl=SSOCheckOnly.

[11] Rowe N., Ilic D. (2015). Rethinking poster presentations at large-scale scientific meetings—Is it time for the format to evolve?. FEBS J..

[12] Brownlie D. (2007). Towards effective poster presentations: An annotated bibliography. Eur. J. Mark..

[13] Bell C., Buckley E.G., Evans P., Lloyd-Jones G. (2006). An evaluation of digital, split-site and traditional formats in conference poster sessions. Med. Teach..

[14] Newsom L.C., Miller S.W., Chesson M. (2021). Use of digital vs printed posters for teaching and learning in pharmacy education. Am. J. Pharm. Educ..

[15] Shin S.J. (2012). Evaluation of electronic versus traditional format poster presentations. Med. Educ..

[16] Powell-Tuck J., Leach S., Maccready L. (2002). Electronic poster presentations in BAPEN—A controlled evaluation. Clin. Nutr..

[17] Venkatesan M., Coskun A.F. (2019). Digital posters for interactive cellular media and bioengineering education. Commun. Biol..

[18] King M., Webster J., Cameron C., Zobel G. (2020). Interactive data-gathering posters as a research tool: A case study assessing public opinion on incorporation of natural behavior into management systems. Animals.

[19] De Simone R., Rodrian J., Osswald B., Sack F.U., De Simone E., Hagl S. (2001). Initial experience with a new communication tool: The ‘Digital Interactive Poster Presentation’. Eur. J. Cardiothorac. Surg..

[20] International Society for Pharmacoeconomics and Outcomes Research. https://www.ispor.org/conferences-education/event/2022/05/15/default-calendar/virtual-ispor-2022.

[21] American Association of Colleges of Pharmacy. https://www.aacp.org/pharmed2020.

[22] Balakrishnan A., Puthean S., Satheesh G., Unnikrishnan M.K., Rashid M., Nair S., Thunga G. (2021). Effectiveness of blended learning in pharmacy education: A systematic review and meta-analysis. PLoS ONE.

[23] Bashir A., Bashir S., Rana K., Lambert P., Vernallis A. (2021). Post-COVID-19 adaptations; the shifts towards online learning, hybrid course delivery and the implications for biosciences courses in the higher education setting. Front. Educ..

[24] Axon D.R., Campbell P., Warholak T. (2019). Student pharmacists’ experiences of teamwork in a quality improvement course. Curr. Pharm. Teach. Learn..

